# Supplementary material for: “I was given PrEP, but had no privacy”: Mystery shopper perspectives of PrEP counseling for adolescent girls and young women in Kisumu County, Kenya
Source: PLoS One. 2024 Aug 19;19(8):e0309075. doi: 10.1371/journal.pone.0309075 (PMC11332929; doi:10.1371/journal.pone.0309075)
Supplement: Attachment — Submitted filename: Response to Reviewers.docx [file pone.0309075.s002.docx]

**Response to Reviewers**

Dear Matt Price and Eduard Sanders,

Thank you for your feedback and time. Below are my responses to your edits for Manuscript: PONE-D-24-01290, “I was given PrEP, but had no privacy”: Mystery shopper perspectives of PrEP counseling for adolescent girls and young women in Kisumu County, Kenya.

Journal Requirements:

1. When submitting your revision, we need you to address these additional requirements.

Please ensure that your manuscript meets PLOS ONE's style requirements, including those for file naming.

Addressed. I reviewed the PDF links and reformatted the title page and main manuscript per PLOS One requirements.

2. Please include a complete copy of PLOS’ questionnaire on inclusivity in global research in your revised manuscript.

Completed and uploaded to submission.

3. Thank you for stating the following financial disclosure:
This study was funded through P.K. by the National Institutes of Health (NIH, R01 HD094630) and M.V. received additional funding by the National Institute of Nursing Research (NINR, T32NR019761).

Please state what role the funders took in the study. If the funders had no role, please state: "The funders had no role in study design, data collection and analysis, decision to publish, or preparation of the manuscript."
If this statement is not correct you must amend it as needed.
Please include this amended Role of Funder statement in your cover letter; we will change the online submission form on your behalf.

Completed and changed in the Cover Letter. Added: "The funders had no role in study design, data collection and analysis, decision to publish, or preparation of the manuscript."

4. In the online submission form, you indicated that your data is available only on request from a third party. Please note that your Data Availability Statement is currently missing [the contact details for the third party, such as an email address or a link to where data requests can be made]. Please update your statement with the missing information.

Amended the online statement with an email and phone number to contact the third party (UW Human Subjects Research Dept.).

5. Please review your reference list to ensure that it is complete and correct. If you have cited papers that have been retracted, please include the rationale for doing so in the manuscript text, or remove these references and replace them with relevant current references. Any changes to the reference list should be mentioned in the rebuttal letter that accompanies your revised manuscript. If you need to cite a retracted article, indicate the article’s retracted status in the References list and also include a citation and full reference for the retraction notice.

Reviewed References and did not need to retract any citations.

Additional Editor Comments:

I have reviewed and have only minor comments. In general, the paper is well written and easy to follow. Two minor suggestions:

Results, first sentence: what is “case 1”, “case 2” etc. I thought there were 8 actors/USPs not 6, is this the number of interviews from each USP? I see from the table this refers to scenarios or scripts. Perhaps this could be better described in the methods?

Did each USP participate in each scenario/case, or did some “specialize” in certain cases, so to speak?

Added: "Two USPs were assigned to each case (some overlapping cases) using scripted commonly occurring PrEP scenarios (Table 1) to seek PrEP services at all 24 facilities." to the Data Collection sub-section.

[Note: HTML markup is below. Please do not edit.]

Reviewers' comments:

Reviewer's Responses to Questions

**Comments to the Author**

1. Is the manuscript technically sound, and do the data support the conclusions?

The manuscript must describe a technically sound piece of scientific research with data that supports the conclusions. Experiments must have been conducted rigorously, with appropriate controls, replication, and sample sizes. The conclusions must be drawn appropriately based on the data presented.

Reviewer #1: Yes

2. Has the statistical analysis been performed appropriately and rigorously?

Reviewer #1: N/A

3. Have the authors made all data underlying the findings in their manuscript fully available?

The [PLOS Data policy](https://nam12.safelinks.protection.outlook.com/?url=https%3A%2F%2Furldefense.com%2Fv3%2F__http%3A%2F%2Fwww.plosone.org%2Fstatic%2Fpolicies.action*sharing__%3BIw!!JmPEgBY0HMszNaDT!v5ILNCHthpIRj0RJiNqSXlh5E7wOL98BOhbmiBqvUypeUTFl6tdsYk3Kyh2iFinkWwqU3XFpgQ_N3RcRoag%24&data=05%7C02%7Cmelissa.vera%40wsu.edu%7C337107f1c17b4c30430908dc5596cea0%7Cb52be471f7f147b4a8790c799bb53db5%7C0%7C0%7C638479353702357332%7CUnknown%7CTWFpbGZsb3d8eyJWIjoiMC4wLjAwMDAiLCJQIjoiV2luMzIiLCJBTiI6Ik1haWwiLCJXVCI6Mn0%3D%7C0%7C%7C%7C&sdata=H2QSPYKuFVZE%2F4Xp%2BqjWCk1Ivu%2FgHuvBk1eVaKy3UGA%3D&reserved=0) requires authors to make all data underlying the findings described in their manuscript fully available without restriction, with rare exception (please refer to the Data Availability Statement in the manuscript PDF file). The data should be provided as part of the manuscript or its supporting information, or deposited to a public repository. For example, in addition to summary statistics, the data points behind means, medians and variance measures should be available. If there are restrictions on publicly sharing data—e.g. participant privacy or use of data from a third party—those must be specified.

Reviewer #1: Yes

4. Is the manuscript presented in an intelligible fashion and written in standard English?

PLOS ONE does not copyedit accepted manuscripts, so the language in submitted articles must be clear, correct, and unambiguous. Any typographical or grammatical errors should be corrected at revision, so please note any specific errors here.

Reviewer #1: Yes

5. Review Comments to the Author

Please use the space provided to explain your answers to the questions above. You may also include additional comments for the author, including concerns about dual publication, research ethics, or publication ethics. (Please upload your review as an attachment if it exceeds 20,000 characters)

**Reviewer #1:** Authors present a large qualitative study of mystery clients who presented as AGYW who were interested to discuss PrEP continuation/prescription. The study was embedded in a large trial involving 24 health facilities. Data were thematically analysed and revealed three themes reflecting on the interactions experienced.

Privacy, attitudes to clients, and 'patient-centered communication' - emerge as main perceived determinants of improved HIV preventive care (i.e. PrEP continuation and initiation).

The paper is well-written reinforces that clinician attitudes impact uptake of prevention products.

The discussion is fairly brief and reiterates the three major findings. I was missing a broader discussion on what it will take to get AGYW to take up daily oral PrEP.

It is clear that improved privacy and client-centred communication is one aspect. There are several other aspects of PrEP programming (e.g. stigma reduction, societal attitudes to sexual activity and PrEP, mental health support, alternative PrEP dispensing approach, role of peer support) that go beyond the mystery clients approach but could be mentioned to contextualise study findings.

The final sentence of the discussion is weak: "Future research is needed that includes a broader sample of non-actor AGYW participants to evaluate of whether provider and health systems interventions to improve these interactions will result in improved uptake and persistence in this priority population."

Especially since authors speak about health systems interventions that have hardly been discussed or put in context.

Small point: how did the sex (gender) of the provider impact interactions?

Thank you for your review and extremely helpful comments. I have added more context to the Discussion and Conclusion sections as stated below.

First paragraph in Discussion; added: "This adds to the broader context in which AGYWs are seeking HIV prevention services where stigma reduction is paramount and changing discriminating attitudes towards HIV prevention and sex are crucial to decreasing HIV transmission. If AGYW can receive better care in the setting they receive care most often (i.e. within the healthcare system among health care providers), it could help with HIV prevention efforts among this population."

Discussion; added ending paragraph: "In the broader context of HIV prevention among AGYW, more is needed: more access points to PrEP, better systems for retention of AGYW to PrEP services, an overall cultural shift toward more understanding and acceptance of AGYW sexual practices and what they do to protect themselves. Retail pharmacies are a promising new addition to PrEP acquisition.^33^ Purchasing PrEP over-the-counter has shown in recent studies to provide the privacy, good communication, and convenience many AGYWs prefer when accessing PrEP. Peer-support groups are another way to potentially increase PrEP intiation among AGYWs.^34^ This model utilizes experienced AGYWs to help PrEP-naïve AGYWs take HIV self-tests and then refers them to PrEP services at clinic. Peer-supported PrEP initiation may be another way to bolster PrEP initiation among AGYWs, but they still need a good experience at the clinic once they get there. Our study reinforces the potential impact that health care providers have among AGYWs accessing HIV prevention."

Conclusion; added: "Further, more research is needed to better contextualize and understand the benefits of retail pharmacy-based PrEP services and peer-supported PrEP programs, both aimed at increasing PrEP access points and retaining more clients on PrEP for younger populations."

6. PLOS authors have the option to publish the peer review history of their article ([what does this mean?](https://nam12.safelinks.protection.outlook.com/?url=https%3A%2F%2Furldefense.com%2Fv3%2F__https%3A%2F%2Fjournals.plos.org%2Fplosone%2Fs%2Feditorial-and-peer-review-process*loc-peer-review-history__%3BIw!!JmPEgBY0HMszNaDT!v5ILNCHthpIRj0RJiNqSXlh5E7wOL98BOhbmiBqvUypeUTFl6tdsYk3Kyh2iFinkWwqU3XFpgQ_NLiutV5Q%24&data=05%7C02%7Cmelissa.vera%40wsu.edu%7C337107f1c17b4c30430908dc5596cea0%7Cb52be471f7f147b4a8790c799bb53db5%7C0%7C0%7C638479353702363920%7CUnknown%7CTWFpbGZsb3d8eyJWIjoiMC4wLjAwMDAiLCJQIjoiV2luMzIiLCJBTiI6Ik1haWwiLCJXVCI6Mn0%3D%7C0%7C%7C%7C&sdata=dz3HoktOy0AgXvVMDJBwNLUC33iWAw4r7HSviD9hMyY%3D&reserved=0)). If published, this will include your full peer review and any attached files.


If you choose “no”, your identity will remain anonymous but your review may still be made public.


**Do you want your identity to be public for this peer review?** For information about this choice, including consent withdrawal, please see our [Privacy Policy](https://nam12.safelinks.protection.outlook.com/?url=https%3A%2F%2Furldefense.com%2Fv3%2F__https%3A%2F%2Fwww.plos.org%2Fprivacy-policy__%3B!!JmPEgBY0HMszNaDT!v5ILNCHthpIRj0RJiNqSXlh5E7wOL98BOhbmiBqvUypeUTFl6tdsYk3Kyh2iFinkWwqU3XFpgQ_NhPeCOXE%24&data=05%7C02%7Cmelissa.vera%40wsu.edu%7C337107f1c17b4c30430908dc5596cea0%7Cb52be471f7f147b4a8790c799bb53db5%7C0%7C0%7C638479353702371463%7CUnknown%7CTWFpbGZsb3d8eyJWIjoiMC4wLjAwMDAiLCJQIjoiV2luMzIiLCJBTiI6Ik1haWwiLCJXVCI6Mn0%3D%7C0%7C%7C%7C&sdata=Z%2B2Qs%2FdsU6FETqSqcsngr3K5mYIL1LIP1T%2BJUyGxKv4%3D&reserved=0).

Reviewer #1: **Yes:** Eduard Sanders
